# Non-Tuberculous Mycobacteria in Respiratory Specimens of Patients with Obstructive Lung Diseases—Colonization or Disease?

**DOI:** 10.3390/antibiotics9070424

**Published:** 2020-07-20

**Authors:** Monika Szturmowicz, Karina Oniszh, Dorota Wyrostkiewicz, Piotr Radwan-Rohrenschef, Dorota Filipczak, Anna Zabost

**Affiliations:** 1 Ist Department of Lung Diseases, National Tuberculosis and Lung Diseases Research Institute, Płocka 26, 01-138 Warsaw, Poland; dw707@wp.pl (D.W.); parawan1970@wp.pl (P.R.-R.); 2 Department of Radiology, National Tuberculosis and Lung Diseases Research Institute, Płocka 26, 01-138 Warsaw, Poland; karina.oniszh@gmail.com; 3 Department of Microbiology, National Tuberculosis and Lung Diseases Research Institute, Płocka 26, 01-138 Warsaw, Poland; d.filipczak@igichp.edu.pl (D.F.); atzabost@wp.pl (A.Z.)

**Keywords:** non-tuberculous mycobacteria, chronic obstructive pulmonary disease, asthma, *Mycobacterium gordonae*, chest computed tomography, body mass index

## Abstract

Non-tuberculous mycobacteria (NTM) are increasingly a cause of human respiratory tract colonization and mycobacterial lung disease (NTM-LD), especially in patients with chronic lung diseases. The aim of the present study was to find the factors predictive of NTM-LD in patients with obstructive lung diseases and NTM respiratory isolates. A total of 839 isolates of NTM, obtained from 161 patients between 2010 and 2020 in a single pulmonary unit, have been retrospectively reviewed. Of these isolates, 73 concerned 36 patients with obstructive lung diseases (COPD-26, asthma-3, COPD/asthma overlap syndrome-7). NTM-LD was recognized according to the American Thoracic Society (ATS) and the Infectious Diseases Society of America (IDSA) criteria in 17 patients, colonization in 19. Lower BMI, elevated body temperature on admission, infiltrative/cavitary lesions on chest CT, and NTM species other than *Mycobacterium gordonae* were the significant predictors of NTM-LD recognition. Based on the above-mentioned predictive factors, an original scoring system was implemented. The diagnostic utility of the scoring system was higher than that of single parameters. We conclude that NTM-LD prediction in patients with obstructive lung diseases and positive respiratory isolates is difficult. A scoring system based on clinical, radiological and microbiological characteristics was capable of facilitating the differential diagnosis, but it needs further validation in a larger study group.

## 1. Introduction

Non-tuberculous mycobacteria (NTM) are common environmental microorganisms, present in water, soil and soil dust [1]. Increasing use of humidifiers, air conditioning, showers and sprinklers may result in greater opportunities for respiratory colonization with NTM present in water aerosols [2].

Incidence rate of NTM respiratory isolation in the United States increased from 8.2/100,000 in 1994 to 16/100,000 in 2014, and concerned three species: *Mycobacterium avium*, *Mycobacterium abscessus*/*chelonae* and *Mycobacterium fortuitum* [3]. Shah et al. noticed the increase of NTM isolates in England, Wales and Northern Ireland from 5.6/100,000 in 2007 to 7.6/100,000 in 2012 [4]. *M. avium* and *Mycobacterium intracellulare* were the predominating species. The increasing rate of *M. avium* isolates between 2000 and 2007 was also noted in Denmark [5].

Accordingly, the growing incidence rate of NTM lung disease (NTM-LD) has been observed in many countries, especially in persons above 60 years of age [4,6,7,8].

The pathogenic effect of NTM on humans depends, among other factors, on the host’s susceptibility to infection. Patients with chronic lung diseases such as chronic obstructive pulmonary disease (COPD), cystic fibrosis, silicosis and past tuberculosis are at greater risk of NTM-LD development due to altered lung structure, chronic inflammation in bronchial airways and impaired mucociliary clearance, which result in prolonged colonization by NTM [1,2,9].

The results of a population study by Andrejak et al. indicated that the risk of NTM-LD increased 15.7-fold in COPD and 7.8-fold in asthma patients, compared to the general population [9]. As COPD affects 8% to 10% of adults in the United States and Europe, it may become the leading cause of susceptibility to NTM-LD in the future [9].

The diagnostic criteria of NTM-LD recognition were published by ATS/IDSA in 2007 [10] and have been reviewed recently by the British Thoracic Society (BTS) [2]. NTM-LD is recognized in patients with new clinical symptoms, radiological changes suggestive of NTM-LD such as infiltrates with cavitation or nodular bronchiectatic form of disease and in whom NTM were cultured once from bronchial washings or twice from separate sputum specimens [2,10].

There are several diagnostic difficulties in applying these recommendations to COPD patients. Clinical symptoms of COPD exacerbation, such as worsening of dyspnea, increased sputum production and elevated body temperature, may be caused by different types of infective pathogens. Such infective exacerbations, not necessarily caused by NTM, are especially common in patients with COPD and bronchiectasis [11,12]. Moreover, positive respiratory cultures for NTM are often obtained with a delay, when clinical response to antibiotics has been already achieved.

The radiological signs of NTM-related disease may be difficult to recognize on a chest X-ray, and chest CT is not an obligatory diagnostic procedure in case of COPD exacerbation.

Nevertheless, NTM-LD worsens life quality and expectancy in COPD patients and thus it should be diagnosed and treated, if possible [13]. The treatment result depends, among others, on the type of mycobacteria and susceptibility to recommended drugs [1]. Macrolides and quinolones, which are often included in the therapy of NTM-LD, are frequently used as antibacterial agents; therefore, there is growing awareness of the possibility of inducing resistance of NTM to these agents in the population.

The aim of the present study was to find the factors predictive of NTM-LD recognition in patients with obstructive lung diseases in whom NTM-positive respiratory specimens have been obtained.

## 2. Results

The retrospective analysis of cultures of sputum and/or bronchial washings obtained in a single pulmonary department between 2010 and 2020 revealed 839 isolates of NTM, obtained from 161 patients. Among those, 73 isolates concerned 36 patients with obstructive lung diseases. Thus, the study group consisted of 36 patients, 26 diagnosed with COPD, 7 with bronchial asthma and 3 with COPD/asthma overlap syndrome. Furthermore, 25 patients have been treated with 400 to 1000 mcg of inhaled corticosteroids, 6 with oral steroids (2 with methylprednisolone 1 and 8 mg/day, and 4 with prednisone 10 to 20 mg/day).

The population characteristics are presented in Table 1.

NTM-LD was recognized in 17 patients, and bronchial colonization with NTM was recognized in 19 patients.

The two groups have been compared in relation to clinical and demographic data (Table 1). No differences between the two groups concerning age, gender, smoking habits, spirometry results and dose of inhaled corticosteroids have been found. Mean BMI was significantly lower in NTM-LD compared to the colonization group (21.9 ± 3.8 vs. 26.8 ± 4.5, respectively, *p* = 0.001). BMI ≤ 22.5 was found in 59% of those with NTM-LD and 21% of the bronchial colonization group, *p* = 0.04. Elevated body temperature on admission was recorded more frequently in NTM-LD compared to the colonization group, 53% vs. 11%, respectively, *p* = 0.02, whereas no differences between the groups concerning the frequency of hemoptysis, dyspnea or purulent expectoration were noted.

**Figure 1:**
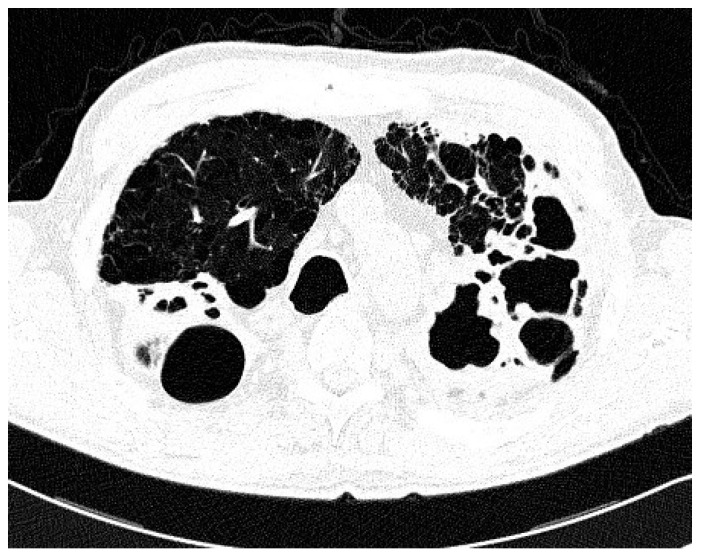
Trans-axial lung window CT scan in patient with non-tuberculous mycobacteria lung disease (NTM-LD) and chronic obstructive pulmonary disease (COPD) shows emphysema and multiple thick-walled cavities in both upper lobes.

**Figure 2:**
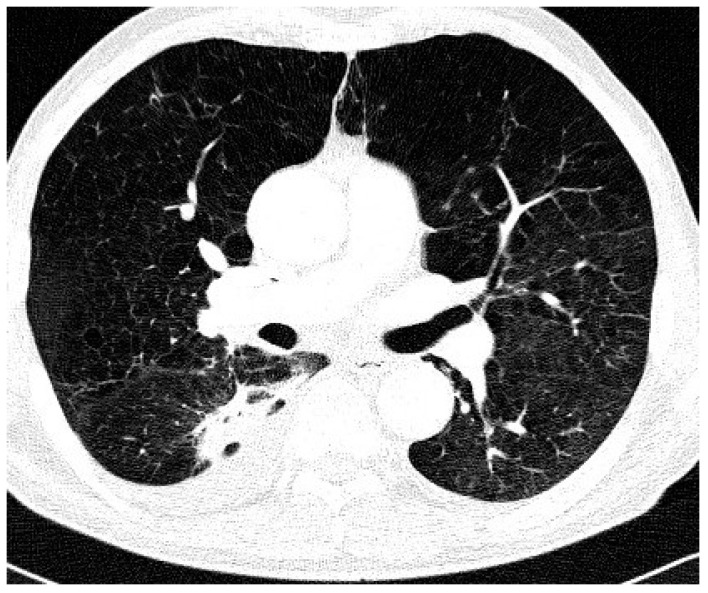
Trans-axial lung window CT scan in patient with NTM-LD and COPD shows diffuse emphysema and cavitating dense infiltrate and pleural thickening in the right apical segment of the lower lobe.

Comparison of the two groups in relation to chest CT appearance (Table 2) revealed that nodular/bronchiectatic changes on chest CT were found with equal frequency in NTM-LD and the colonization group (47%). The infiltrative/cavitary form of disease was found in 35% of the NTM-LD group and none of the patients in the colonization group. The examples of infiltrative/cavitary lesions on chest CT were presented on Figure 1 and Figure 2.

Non-specific lesions were described more often in the colonization group than in NTM-LD (57% vs. 18%, respectively). The above-mentioned differences were significant (*p* = 0.03).

According to microbiological data, *M. avium* was cultured in 39% of the examined population, more frequently in NTM-LD than in the colonization group (60% vs. 26%, respectively, difference not significant) (Table 3). *M. gordonae* was cultured only in patients belonging to the colonization group; other NTM species were found with the same frequency in both groups. The difference between the distribution of *M. gordonae* and the remaining NTM species was significant, *p* = 0.018. Co-infection with NTM and Gram-negative bacteria was found in 39% of patients, with NTM and *Aspergillus fumigatus* in 17%.

Univariate analysis (Table 4) revealed that low BMI, elevated body temperature on admission, type of NTM other than *M. gordonae*, and the presence of infiltrative-cavitary form of disease on chest CT were the significant indicators of NTM-LD.

Based on these data, we proposed an algorithm of NTM-LD probability calculation in patients with obstructive lung diseases: identification of *M. gordonae*—minus 3 points, infiltrative-cavitary type of disease on chest CT—3 points, non-specific chest CT lesions—minus 1 point, BMI ≤ 22.5—2 points and elevated body temperature on admission—1 point. Median score in NTM-LD patients was 2 points (range 0 to 6 points) and in the colonization group—minus 1 point (range −4 to 2 points), *p* = 0.001. The optimal cut-off was 1 point (94% sensitivity, 84% specificity, 82% PPV, 94% NPV). Probability of NTM-LD was increased by 5.75 times in patients scoring 1 point or higher (Table 4).

## 3. Discussion

Growing incidence of NTM-LD in obstructive lung diseases requires an awareness among medical staff concerning the probability of NTM infection. One of the predisposing factors is treatment with inhaled corticosteroids (ICS) [9,14]. ICS are the first-line therapy in asthma patients, but in COPD they are indicated only in those who are highly symptomatic, with increased number of hospitalizations (>2/per year) and severe bronchial obstruction [15]. In our study group, most patients presented with moderate or severe bronchial obstruction, and median FEV1% pred. was 50%. No significant differences concerning the spirometry results have been found between the NTM-LD and the colonization group. ICS have been used by 65% of COPD patients with positive NTM isolates, mostly in high doses (800 to 1000 mcg/day). The same observation was made by Andrejak et al. who documented ICS therapy in 64% of COPD patients [9]. The authors concluded that high doses of ICS had also been prescribed in those COPD patients who had no indications for such therapy [9].

The other risk factor of NTM isolation is the recognition of COPD-bronchiectasis overlap syndrome [11,12]. Gatheral et al. documented the presence of bronchiectasis in 69% of COPD patients, and frequent coinfection with *Pseudomonas aeruginosa* and NTM in this group of patients [11]. In our study group, bronchiectasis was recognized in 44% of patients with obstructive lung diseases, with the same frequency in NTM-LD and colonization groups.

The analysis of clinical symptoms in our study group revealed that most patients suffered from dyspnea (72%) with increased expectoration (47%). Nevertheless, no differences between NTM-LD and colonization group were noted. Although 22% of the patients noted hemoptysis, this symptom was not indicative of NTM-LD, either. The only significant difference concerned elevated body temperature that was noted in 53% of the NTM-LD group and 11% of the colonization group.

The other factor indicative of NTM-LD was low BMI. Mean BMI values were 26.8 in the colonization group and 21.9 in NTM-LD, *p* = 0.001. Optimal BMI cut-off was calculated as 22.5, the values equal or lower than 22.5 were found in 59% of the NTM-LD group and 21% of the colonization group. Low BMI was also a factor predictive of NTM-LD in the study of Huang et al. [16].

The analysis of chest CT revealed the presence of nodular bronchiectatic form of disease in 47% of patients with NTM respiratory isolates. Nevertheless, this form of chest CT disease was not indicative of NTM-LD. The demonstration of infiltrative/cavitary disease was pathognomonic for NTM-LD, but it was diagnosed in 35% of patients with NTM-LD only. The data from the literature indicate that in the patients infected with *M. avium*, the presence of infiltrative/cavitary form of NTM-LD was suggestive of a more aggressive type of disease with worse life expectancy [17]. Nevertheless, no data have been published concerning the prognostic role of infiltrative/cavitary disease in COPD patients.

Post-tuberculous lung lesions were frequent (53%), but they were not identified as a risk factor of NTM-LD in the present study group of patients with obstructive lung diseases. Increased susceptibility to NTM-LD in patients with past tuberculosis was documented in our previous report, but it concerned patients with all types of chronic lung disease [18]. Susceptibility to NTM colonization of post-tuberculous lesions may concern primarily *M. kansasii* [19,20], which was not a frequent NTM type in the present study group.

Analysis of microbiologic data revealed that *M. gordonae* was not pathogenic, as it was identified only in the colonization group. Lack of pathogenic effect of *M. gordonae* was also documented by Adzic et al., who analyzed the clinical significance of positive respiratory NTM isolates in Serbia [19].

*M. avium* was the most frequent NTM species noted in 39% of our study group, 60% of the NTM-LD group and 26% of the colonization group, although the difference was not significant. *M. avium* was also the most frequent NTM type identified in the study of American veterans hospitalized due to COPD exacerbation [21], as well as in the study of Huang et al. concerning COPD patients diagnosed in Taiwan [16].

Univariate analysis revealed that risk of NTM-LD in our study group was increased in the case of elevated body temperature, infiltrative/cavitary form of chest CT disease, low BMI and NTM-type other than *M. gordonae*.

Taking into consideration the results of risk factor analysis, we proposed clinical scoring, improving the prediction of NTM-LD in obstructive lung diseases. The proposed scoring system improved the clinical prediction of NTM-LD recognition in patients with obstructive lung diseases. The score of 1 point or more was combined with an over 5-fold increase of NTM-LD probability. Nevertheless, the scoring system requires validation in larger groups of patients with obstructive lung diseases and positive NTM isolates.

In summary, the diagnosis of NTM-LD in obstructive lung diseases is difficult and should be based on analysis of clinical data, results of chest CT and identification of NTM species.

The indications for ICS use should be verified in COPD patients with frequent exacerbations, as it may be responsible for NTM colonization. Periodic chest CT examination and sputum cultures for mycobacteria should be mandatory in patients with frequent exacerbations of obstructive lung diseases.

## 4. Materials and Methods

Sputum or bronchial washings obtained during fiber-optic bronchoscopy were decontaminated with sodium hydroxide and *N*-acetyl-L-cysteine. Smears for acid-fast bacilli (AFB) of the processed specimens were stained with auramine fluorochrome and examined by standard procedures. Fluorochrome stain-positive smears were confirmed by the Ziehl-Neelsen method. Genetic tests were performed by BD ProbeTec *M. tuberculosis* complex (DTB).

The strains were cultured on solid media: egg-based Löwenstein-Jensen medium and in automated system MGIT (Becton Dickinson, Franklin Lakes, NJ, USA). The identification of the species was performed with molecular test GenoType CM (Hain Lifescience, Nehren, Germany), version 1.0 and 2.0. The procedure consisted of three steps: DNA isolation, DNA amplification using primers labelled with biotin and DNA reverse hybridization. The method allowed for the identification of *M. tuberculosis* complex as well as 14 clinically relevant NTM species.

Clinical data have been obtained from hospital electronic databases. Age, gender, smoking habits, BMI, coexisting diseases, as well as signs and symptoms of obstructive lung disease exacerbation were recorded.

Chest CT scans, performed according to various protocols depending on clinical context of admission, have been retrospectively reviewed by an experienced radiologist and pulmonologist blinded to the clinical diagnosis. Lung disease that might be attributed to NTM-LD has been classified as nodular/nodular-bronchiectatic lesions or infiltrations/cavitation. Other forms of lung pathology, such as solitary nodules, areas of parenchymal fibrosis with bronchiectasis, have been coded as non-specific lesions. Additionally, chest CT scans were reviewed for signs of past tuberculosis. Post-tuberculous lung lesions were defined as calcified nodules with lymph-node calcifications (primary syndrome) as well as fibrotic foci localized in the upper lobes.

Statistical analysis was performed with R environment [22]. The normality of distribution of continuous variables was checked with Kolmogorov-Smirnov test. Student *t* test or Mann-Whitney rank sum test with continuity correction (depending on normality of distribution) were used for the comparison of continuous variables in the NTM-LD and colonization groups. Univariate logistic regression analysis concerning prediction of NTM-LD was performed. *p*-value of <0.05 was considered statistically significant.

The study was conducted in accordance with the Declaration of Helsinki. The project was approved by the Ethics Committee of the National Institute of Tuberculosis and Lung Diseases, identification code: 9/2015.

## Figures and Tables

**Table 1:** Population characteristics in 36 patients with obstructive lung diseases and non-tuberculous mycobacteria (NTM) respiratory isolates.

Factor	Total Population No. 36	NTM-LD No. 17	Colonization No. 19	*p*
Gender Males/Females	15/21	7/10	8/11	0.95
Age, mean (SD)	65, 9 (11,6)	65, 2 (11,1)	66, 3 (12,2)	0.85
BMI, mean (SD)	24, 5 (4,8)	21, 9 (3,8)	26, 8 (4,5)	0.001
≤22.5 No (%)	14 (39)	10 (59)	4 (21)	0.04
Smoking/pack-years median (range)	20 (0–80)	25 (0–50)	20 (0–80)	0.22
Inhaled CS No. (%)	25 (69)	12 (71)	13 (68)	0.82
Oral CS No. (%)	5 (14)	2 (12)	3 (16)	0.89
Spirometry (median, range)				
FEV1%/FVC	0.51 (0.3–0.86)	0.45 (0.3–0.75)	0.56 (0.41–0.86)	0.09
FEV1% pred.	50 (22–123)	45 (22–123)	57 (34–110)	0.41
FVC% pred.	77.5 (49–129)	(52–129)	(49–106)	0.48
Weight loss > 5% No (%)	6 (17)	4 (24)	2 (11)	0.38
Elevated body temperature (>38 °C) No. (%)	11 (31)	9 (53)	2 (11)	0.02
Dyspnea No. (%)	26 (72)	13 (77)	13 (68)	0.87
Haemoptysis No. (%)	8 (22)	4 (24)	4 (21)	0.82
Expectoration No. (%)	17 (47)	7 (41)	10 (53)	0.72
Bronchiectasis No. (%)	16 (44)	7 (41)	11 (58)	0.5
Hypothyreosis No. (%)	7 (19)	3 (18)	4 (21)	0.87
Past neoplastic disease No. (%)	6 (17)	4 (24)	2 (11)	0.55
Diabetes No. (%)	6 (17)	5 (29)	1 (5)	0.14

BMI—body mass index, CS—corticosteroids, FEV1—forced expiratory volume in one second, FVC—forced vital capacity, FEV1/FVC—Tiffeneau-Pinelli index.

**Table 2:** Results of chest CT examination in 36 patients with obstructive lung diseases and with positive NTM respiratory isolates.

Type of Chest CT Changes	Total Population No. 36	NTM-LD No. 17	Colonization No. 19	*p*
Nodular/bronchiectasis No. (%)	17 (47)	8 (47)	9 (47)	0.03
Infiltrations/cavities No. (%)	6 (17)	6 (35)	0 (0)	
Non-specific No. (%)	13 (36)	3 (18)	10 (57)	
Post tuberculous lesions No. (%)	19 (53)	10 (59)	9 (47)	0.72

**Table 3:** Results of microbiological examination in 36 patients with obstructive lung diseases.

Species	Total Population No. 36	NTM-LD No. 17	Colonization No. 19	*p*
*M. avium*	14 (39)	9 (60)	5 (26)	*M. gordonae* versus remaining species 0.018
*M. gordonae*	7 (19)	0 (0)	7 (37)	
*M. kansasii*	6 (17)	3 (18)	3 (16)	
*M. xenopi*	6 (17)	3 (18)	3 (16)	
*M. fortuitum*	2 (6)	1 (6)	1 (5)	
*M. intracellulare*	1 (3)	1 (3)	0 (0)	
*P. aeruginosa*	5 (14)	1 (3)	4 (21)	0.4
Other Gram (-)	9 (25)	4 (24)	5 (26)	0.85
*A. fumigatus*	6 (17)	3 (18)	3 (16)	0.77
*Candida sp.*	9 (25)	5 (29)	4 (22)	0.85

**Table 4:** Risk of NTM-LD in 36 patients with obstructive lung diseases based on clinical, microbiological and radiological characteristics.

Factor	HR	95%CI	*p*
BMI	3.55	2.84–4.12	0.001
BMI < 22.5	2.45	1.16–3.85	0.002
Elevated body temperature	3.02	2.24–4.96	0.005
CT infiltrative-cavitary type	3.38	2.54–5.38	0.002
NTM other than *M. gordonae*	3.06	2.92–4.45	0.004
Probability scoring	5.75	4.19–6.64	0.001
